# Pituitary as a Source of HCG: Residual Levels After Bilateral Testicular Tumor Removal

**DOI:** 10.1177/2324709619841414

**Published:** 2019-04-22

**Authors:** Richard Santen, Farhad Hasan, Katie Thoren, Azeez Farooki

**Affiliations:** 1 University of Virginia Health Sciences System, Charlottesville, VA, USA; 2 Memorial Sloan Kettering Cancer Center, Cornell-Weil School of Medicine, New York, NY, USA

**Keywords:** HCG, pituitary secretion, testicular tumor

## Abstract

*Context*. Challenging clinical scenario in which elevated β-human chorionic gonadotropin (HCG, subsequently termed HCG) levels suggested occult tumor metastases after removal of bilateral testicular cancers and metastases from them and as well as after chemotherapy. *Case Report*. A 22-year-old male, post excision of bilateral testicular tumors, who had no imaging or clinical evidence of residual tumor but an elevated HCG raising the question of the presence and location of occult tumor metastases. *Clinical Questions*. Does luteinizing hormone (LH) cross-react with HCG in current assays? What levels of testosterone and estradiol are necessary to suppress LH and follicle-stimulating hormone (FSH) in a male patient with bilateral orchiectomy, and therefore lacking inhibin? Does the pituitary secrete HCG and under what circumstances? *Assessment*. Current HCG assays no longer cross-react with LH as did prior assays, but the presence of heterophile antibodies and other factors such as biotin can still cause false positive HCG levels. In the chronic post-orchiectomy state, the pituitary is relatively resistant to LH and FSH suppression by testosterone. The pituitary secretes HCG in very small amounts unless interruption of negative feedback results in high LH and FSH whereupon HCG levels become elevated. *Clinical Conclusion*. A GnRH antagonist suppressed both LH and HCG in this patient indicating that the elevated HCG was secreted by the pituitary and not by occult tumor metastases. Further credence for this conclusion resulted from the lack of a progressive increase in HCG levels over a 4-year period of follow-up and from no evidence of metastatic tumors on serial imaging.

## Introduction

The pituitary gland synthesizes both luteinizing hormone (LH) and follicle-stimulating hormone (FSH), which are composed of common α but unique β subunits.^1^ Both molecular and clinical data suggest that the pituitary also synthesizes small amounts of human chorionic gonadotropin (HCG), which contains its own β subunit.^2-11^ In women lacking functioning ovaries, in whom the levels of LH and FSH are high, the presence of circulating HCG of pituitary origin has been demonstrated.^2,9-11^ These data suggest that pituitary HCG is under the control of gonadotropin-releasing hormone (GnRH) and that when negative feedback is interrupted and GnRH increases, the pituitary releases a sufficient amount of HCG and/or its β subunit to be detectable in the serum. This phenomenon has been clearly demonstrated in agonadal women, but not previously in men.

In this report, we present the case of a man with bilateral testicular cancers who had no residual tumor based on imaging after bilateral orchiectomy, chemotherapy, and resection of abdominal masses. However, residual HCG was detected at a time when the LH and FSH levels were elevated. The clinical dilemma was whether the HCG represented a small amount of residual testicular tumor, production of HCG from the pituitary or from a tumor of separate tissue origin. Suppression of LH with a GnRH antagonist caused complete suppression of serum HCG to undetectable levels, suggesting that the residual HCG was from the pituitary. A 4-year follow-up of the patient indicated no recurrence, *in the face of persistently elevated HCG levels*, providing further support that the HCG was not from the testicular tumors, which are known to be rapidly progressive.

Several interesting questions arose in the evaluation of this patient: the possibility that LH and HCG cross-react; the lack of inhibitory effect of inhibin (which is absent after bilateral orchiectomy) on secretion of LH and FSH; the relative resistance of LH and FSH suppression to testosterone during a chronic post-orchiectomy state; and the possibility of hypometabolism of testosterone resulting in high testosterone levels with standard replacement doses. The evaluation of this patient provided several important pieces of information helpful for endocrinologists in evaluating patients with HCG producing testicular tumors and the hormonal effects of bilateral orchiectomy.

## Materials and Methods

Routine blood analysis was performed in the Clinical Chemistry Laboratory at Memorial Sloan Kettering Cancer Center. All measurements were made in serum. HCG and cortisol were measured using the Tosoh AIA 2000 immunoassay analyzer. The HCG assay measures intact HCG, the HCG-β subunit, and hyperglycosylated forms. FSH, LH, prolactin, thyroid stimulating hormone (TSH), and free T4 were measured using the Siemens Advia Centaur XP immunoassay analyzer. Testosterone and SHBG were measured on the Abbott Architect I2000. Serum samples were also sent out to reference laboratories for testing. FSH, LH, and ACTH levels were measured at Esoterix Reference Laboratory, a subsidiary of LabCorps. Inhibin A, inhibin B, total testosterone, and free testosterone (by equilibrium dialysis) were measured at Quest Diagnostics. GnRH was measured by Inter Science Institute. Imaging studies were performed at Memorial Sloan-Kettering Hospital and included serial computed tomography (CT) scans of the abdomen and pelvis (performed to evaluate for testicular tumor metastases), chest X-rays, and a magnetic resonance imaging (MRI) of the sella (to rule out gonadotropinoma). The analysis of tumor tissue pathologically utilized standard techniques employed at the Memorial Sloan-Kettering Hospital.

## Patient Presentation

An 18-year-old white male was diagnosed with a 1.3 cm testicular tumor in July 2012. Pathology revealed a mixed, non-seminomatous germ cell tumor with elements of teratoma, embryonal carcinoma, and yolk sac; the dominant component of the tumor was embryonal. He was treated with orchiectomy in August 2012 and then underwent surveillance. In October 2012, he was found to have lymphatic enlargement on CT scan. In November 2012, chemotherapy was initiated with bleomycin, etoposide, and cisplatin. Follow-up CT scan showed an enlarged cystic inter-aorto-caval mass suspicious for teratoma. In January 2013, retroperitoneal lymph node dissection found 3/10 positive nodes, which were resected. Pathology showed pure teratoma. HCG levels on 2 occasions thereafter (ie, on January 17, 2013, and January 23, 2013) were undetectable. Later, the HCG levels became positive and gradually rose from 2.9 to 4.3 mIU/mL between July 24, 2013, and April 14, 2014 (Table 1). On April 24, 2014, a mass was felt on palpation of the left testicle, which was confirmed on ultrasound imaging. Accordingly, a left orchiectomy was performed on April 24, 2014. The pathology was reported as mixed germ cell tumor containing embryonal carcinoma, yolk sac tumor, mature teratoma, and immature teratoma with the dominant tumor type embryonal carcinoma (50%). Preoperatively his LH was 1.2 mIU/mL, his FSH 3.8 mIU/mL, and testosterone 435 ng/dL. He was then started on intramuscular testosterone injections followed by testosterone gel. Postoperatively his HCG was found to be elevated at 4.2 mIIU/mL and testosterone levels exceeded 1000 ng/dL. Serial HCG, LH, FSH, and testosterone levels are shown in Table 1. Furthermore, gonadotropin assays were performed by Esoterix laboratory (subsidiary of Labcorps) and found to be persistently elevated. No residual tumor was found on extensive imaging including serial CT scans of the abdomen and pelvis and also chest X-rays. The key question was whether the patient had a residual tumor lurking somewhere or whether the HCG was somehow falsely elevated.

**Table 1.** Hormone Levels Post-Bilateral Orchiectomy.

	May 2014	June 2014	July 2014	August 2014	September 2014	October 2014	November 2014
β-HCG^a^	<2.0	2.7, 3.3, and <2.0	<2.0	<2.0	2.6	3.5 and 4.2	3.9
Testosterone^b^		1476	141	1864		205	1710
LH^a^		40	38		62	54	52
FSH^a^		83			101	89	95
Testosterone administration	300 mg; IM × 1		40.5 mg by gel daily	20.5 mg by gel daily	20.5 mg by gel daily	40.5 mg by gel daily	40.5 mg by gel daily

Abbreviations: HCG, human chorionic gonadotropin; LH, luteinizing hormone; FSH, follicle-stimulating hormone,

a HCG, FSH, and LH units are mIU/mL.

b Testosterone units are ng/mL.

## Comprehensive Patient Evaluation

The clinical evaluation involved several apparent dilemmas. First was the question of whether the HCG could have been artificially elevated as a function of cross-reactivity with LH. The laboratory specialist at Sloan Kettering Cancer Center, Dr Martin Fleisher, was contacted to determine the level of cross-reactivity between the LH and HCG. The HCG assay in question (a 2-site immunometric assay on the Tosoh AIA 2000 analyzer) measures both free and total HCG. This assay does not cross-react with LH. The sample was sent to Esoterix laboratory (a subsidiary of LabCorp), which found a persistent elevation in HCG with the Roche ECLIA assay (also measures intact HCG and its β-subunit) and also reported that their assay did not cross-react with LH.

The next question was whether the positive HCG might have been due to heterophile antibodies or other factors, which can falsely elevate HCG.^12-14^ To assess this possibility, the sample was incubated with heterophilic blocking tubes (Scantibodies Laboratory, Inc) to remove potential interference from heterophile antibodies. Results after incubation with these tubes did not suggest that heterophile antibodies were present. In addition, serial dilution of the serum was linear, suggesting that the original HCG result was not due to a false positive interference.

It was surprising that the LH and FSH levels were continually elevated in the patient even though the administered testosterone resulted in high levels (1864 and 1476 ng/dL) in the face of normal SHBG levels (21.2 nM/L; normal 13.3-89.5 nM/L). Several repeat testosterone levels were also high (Table 1) and the estradiol level was 39. We reasoned that he was either taking more testosterone than was prescribed or his metabolic clearance rate was very low. Careful history and discussion with his family led to the conclusion that excessive administration of testosterone utilization was very unlikely. It is not well recognized that the metabolic clearance rates of testosterone are highly variable and a substantial number of men have low metabolic clearance rates.^15^ By inference from an understanding of physiology and an anecdotal observation in a patient by one of the authors (RJS), standard replacement doses of testosterone will cause high testosterone levels in orchiectomized men if the metabolic clearance rate is low. Accordingly, low metabolic clearance rate provided the most likely cause of high testosterone levels.

Another possibility that would explain high LH and FSH in the face of high testosterone levels was androgen resistance. This possibility appeared unlikely since the patient had undergone normal puberty, had no history of gynecomastia, a normal estradiol level, and no hypospadias. Another rare possibility to explain high gonadoptropins would be a pituitary gonadotropin producing tumor.^16^ While these are relatively rare, gonadotropin-producing tumors secrete FSH predominantly but LH as well. Prolactin, thyroid function, cortisol, and ACTH were completely normal. An MRI of the sella turcica was obtained, which was normal and made the possibility of a pituitary tumor secreting HCG highly unlikely.

As often occurs with unexplained findings, one needs to consider a number of options as explanations. Our clinical thinking involved the role of bilateral orchiectomy on the ability of androgens to suppress LH and FSH. Since both testes were surgically excised, the patient would not be secreting inhibin, and for that reason, the LH and FSH might not be suppressed normally with even a high level of testosterone. It is well known that in postmenopausal women, who have low inhibin levels, LH and FSH are not suppressed when the women are given replacement estradiol and a progestogen. Review of the literature and personal consultation with the inhibin experts, Henry Burger, David DeKretser, Peter Snyder, and Tony Plant (personal communications), suggested that LH is only minimally affected by inhibin in a negative feedback fashion.^17^ Of particular note, data from Plant et al and Resko et al in monkeys demonstrated resistance to LH suppression after a chronic period after orchiectomy^18-20^ and Winter and colleagues reported this in hypogonadal men.^21^

In patients with cancer, the family often becomes deeply involved. The patient’s mother carefully questioned whether the appropriate diagnostic information had been obtained. In this case, she was very concerned that a lurking, but treatable tumor might be present, as indicated by the persistently elevated HCG. She separately contacted several experts in the area of HCG (see penultimate paragraph below). As many types of tumor secrete HCG,^22-24^ it was suggested that the patient may have harbored a very small tumor not large enough to be picked up on extensive imaging. A similar case had been reported by Rudnick and O’Dell several years ago.^25^ An occult tumor was considered possible, but to assess this, it would be necessary to follow for some time to look for signs and symptoms and then re-image. On the other hand, a little known fact, pointed out by Dr Glenn Braunstein (personal communication), was that the pituitary secretes HCG, but in very small amounts, about 1% of the total gonadotropin. When LH is very high, the HCG also goes up due to increased pituitary secretion. Studies evaluating this in postmenopausal women and during the menstrual cycle have been published.^26,27^

For this reason, the GnRH antagonist, degarelix was given to suppress pituitary secretion of LH and FSH.^28^ This suppressed LH levels from 53.7 to 2.2 ng/mL and HCG from 6.5 ng/mL to <2.0. Review of the literature uncovered reports in which HCG was elevated due to pituitary production of HCG in postmenopausal women and which was suppressed with a GnRH antagonist.^2^ Subsequent to demonstrating an undetectable HCG after degarelix, the GnRH antagonist, in our patient it was assumed that the elevation of HCG was due to pituitary secretion and not to an occult tumor. The patient and his mother were much relieved by this information. During a follow-up of an additional 4 years, the HCG remained slightly elevated but stable and the patient experienced no evidence of recurrent tumor (Supplemental Table; available online).

## Discussion

Several lessons emerge from this prismatic case, which are supported by the clinical data and review of the literature. (1) The pituitary makes HCG^2-6,10,11^ but this is only observed clinically when pituitary gonadotropin production is high. (2) In men with bilateral orchiectomy, the LH and FSH levels may not be suppressed when exogenous testosterone is administered,^18,19^ when this occurs after chronic orchiectomy. (3) Recent assays no longer exhibit cross-reactivity between LH and HCG,^29,30^ but heterophile antibodies, biotin, and other substances can interfere. (4) There are a number of tumors that make HCG^22,23^ as reviewed several years ago by Glenn Braunstein et al. (5) The metabolic clearance rates of testosterone are variable,^15^ and a low metabolic clearance rate will result in a high testosterone level in men with no testes when given a standard dose of testosterone. (6) HCG levels can be elevated in postmenopausal women.^26,27^ One caveat in interpreting the data, as pointed out by an expert in the field (personal communication, William Crowley), is that extragonadal tumors secreting HCG might in fact contain GnRH receptors. Under these circumstances, the GnRH antagonist might have suppressed HCG produced by tumor rather than by the pituitary. While this is a possibility, the long-term follow-up of the patient in which the HCG did not increase over a 4-year period suggest that the pituitary was in fact the source of HCG (Supplemental Table).

The literature has not emphasized in detail the role of the metabolic clearance rate of testosterone and maintaining testosterone concentrations in men with bilateral orchiectomy. Careful studies by one of the coauthors (RJS) showed metabolic clearance rates in a series of normal men of Chinese and Caucasian origin and demonstrated a 5- to 8-fold change in metabolic clearance rate of testosterone from the lowest to the highest.^15^ These studies further showed that a low metabolic clearance rate is associated with a reduced production rate of testosterone, which would maintain the testosterone level normal. If the current patient has a low metabolic clearance rate in the absence of testes, a standard dose of testosterone replacement would result in a high level of circulating testosterone. We believe without firm proof that this is probably the explanation for the high levels of testosterone during testosterone replacement in this patient.

In the early days of radio-immunoassay, the available techniques demonstrated cross-reactivity between LH and HCG. This is not the case with the assays currently available and we could rule out cross-reactivity in this patient.^24^ However, heterophile antibodies and other substances can interfere with HCG assays, but in our patient results from serial dilution of the serum and incubation with heterophile blocking tubes suggest that the elevated HCG levels were not due to interference.^13,29^

Rapid electronic communications facilitate the utilization of experts in the management of difficult or confusing clinical cases. The evaluation of this patient represents an excellent example of this phenomenon. When various issues were not resolved, valuable advice was obtained from Drs Peter Snyder, Glenn Braunstein, Tony Plant, David DeKretser, Henry Burger, William Crowley, and an anonymous expert from the Endocrine Society participating in the *Endocrine Cases* consult program. The suggestion that the HCG represented pituitary secretion came from Dr Glenn Braunstein.

## In Summary

Prismatic cases often provide unique information and it would appear that the secretion of HCG by the pituitary in this patient was a rare but very important finding. Demonstration of suppression of HCG with the GnRH antagonist provided highly comforting information to the patient’s family that a small amount of residual HCG producing choriocarcinoma was not lurking in the patient.

## Supplemental Material

Supplementary_table – Supplemental material for Pituitary as a Source of HCG: Residual Levels After Bilateral Testicular Tumor Removal Click here for additional data file. Supplemental material, Supplementary_table for Pituitary as a Source of HCG: Residual Levels After Bilateral Testicular Tumor Removal by Richard Santen, Farhad Hasan, Katie Thoren and Azeez Farooki in Journal of Investigative Medicine High Impact Case Reports
