# Supplementary material for: Pituitary as a Source of HCG: Residual Levels After Bilateral Testicular Tumor Removal
Source: J Investig Med High Impact Case Rep. 2019 Apr 22;7:2324709619841414. doi: 10.1177/2324709619841414 (PMC6480980; doi:10.1177/2324709619841414)
Supplement: Supplementary_table – Supplemental material for Pituitary as a Source of HCG: Residual Levels After Bilateral Testicular Tumor Removal [file Supplementary_table.pdf]

Supplementary Table

Date	B-HCG	FSH	LH	testosterone
4-23-2015	6.5	93.9	53.7	1080
6-25-2015	< 2	4.6	0.4	786
8-20-2015	< 2	8.4	1.8	647
10-22-2015	< 2	26.8	11.3	1575
3-17-2016	5.8	80.3	49.5	529
6-1-2016	6.6	97.3	59.8	1384
8-11-2016	3.5		42.5	240
3-20-2017	4.1	78.0	39.3	1311
10-26-2017	4.1			
2-1-2018	4.0	61.2	26.6	567
6-7-2018	2.4			
10-11-2018	3.4			

B-HCG nl < 2.2 mIUI/ml; FSH nl 1-18 mIU/ml; LH nl 2.0-9.0 mIU/ml; testosterone nl 221-716 ng/dl
